# Retracted: Fructose Diet-Induced Skin Collagen Abnormalities Are Prevented by Lipoic Acid

**DOI:** 10.1155/2022/9808943

**Published:** 2022-04-19

**Authors:** Journal of Diabetes Research

Journal of Diabetes Research

Journal of Diabetes Research has retracted the article titled “Fructose Diet-Induced Skin Collagen Abnormalities Are Prevented by Lipoic Acid,” [1], due to concerns with images contained within the manuscript as noted on PubPeer [2]. There appears to be a duplication of several bands both within and between the SDS gel patterns in Figures 3(a) and 3(b). Additionally, there appears to be multiple duplications and inconsistencies in the background of the gels within and between both figures.

The authors responded to explain that the similarities between the gels could be because the same set of collagen proteins were screened, but in different fractions. The running patterns of enriched protein bands and residual protein bands are not discrepant within the lane, which the authors considers confirmation that these are accurate images. Additionally, the authors noted that most of the similarities pointed out are artefacts identified by software when the contrast of the picture is altered, and researchers routinely performing SDS page and Western blotting would agree that such things occur commonly.

The authors were unable to provide the original images, which they state is due to the length of time since the experiment was conducted, and were unable to satisfy the concerns of the editorial board. The article is therefore being retracted due to concerns with the reliability of the data. The authors agree to the retraction.
